# CXCR3 modulates glial accumulation and activation in cuprizone-induced demyelination of the central nervous system

**DOI:** 10.1186/1742-2094-11-109

**Published:** 2014-06-16

**Authors:** Marius Krauthausen, Simon Saxe, Julian Zimmermann, Michael Emrich, Michael T Heneka, Marcus Müller

**Affiliations:** 1 Department of Neurology, Universitätsklinikum Bonn, Sigmund-Freud-Str. 25, D-53105 Bonn, Germany

**Keywords:** CXCR3 signaling, cuprizone model, microglia, CXCR3-/-, CNS inflammation

## Abstract

**Background:**

The functional state of glial cells, like astrocytes and microglia, critically modulates the course of neuroinflammatory and neurodegenerative diseases and can have both detrimental and beneficial effects. Glial cell function is tightly controlled by cellular interactions in which cytokines are important messengers. Recent studies provide evidence that in particular chemokines are important modulators of glial cell function. During the course of CNS diseases like multiple sclerosis or Alzheimer’s disease, and in the corresponding animal models, the chemokines CXCL9 and CXCL10 are abundantly expressed at sites of glial activation, arguing for an important role of these chemokines and their corresponding receptor CXCR3 in glial activation. To clarify the role of this chemokine system in glial cell activation, we characterized the impact of CXCR3 on glial activation in a model of toxic demyelination in which glial activation without a prominent influx of hematogenous cells is prototypical.

**Methods:**

We investigated the impact of CXCR3 on cuprizone-induced demyelination, comparing CXCR3-deficient mice with wild type controls. The clinical course during cuprizone feeding was documented for five weeks and for the subsequent four days withdrawal of the cuprizone diet (5.5 weeks). Glial activation was characterized using histological, histomorphometric and phenotypic analysis. Molecular analysis for (de)myelination and neuroinflammation was applied to characterize the effect of cuprizone on CXCR3-deficient mice and control animals.

**Results:**

CXCR3-deficient mice displayed a milder clinical course during cuprizone feeding and a more rapid body weight recovery after offset of diet. In the CNS, CXCR3 deficiency significantly attenuated the accumulation and activation of microglia and astrocytes. Moreover, a deficiency of CXCR3 reduced the expression of the microglial activation markers CD45 and CD11b. Compared to controls, we observed a vast reduction of RNA levels for proinflammatory cytokines and chemokines like *Ccl2*, *Cxcl10*, *Tnf* and *Il6* within the CNS of cuprizone-treated mice. Lastly, CXCR3 deficiency had no major effects on the course of demyelination during cuprizone feeding.

**Conclusions:**

The CXCR3 chemokine system is critically involved in the intrinsic glial activation during cuprizone-induced demyelination, which significantly modulates the distribution of glial cells and the local cytokine milieu.

## Background

Glial cells are essential for neuronal function and homeostasis of the CNS [1-6]. In addition, glial cells are key participators during pathological changes within the diseased brain [7-10]. Both microglia and astrocytes can be rapidly activated through mechanisms like pattern recognition receptors detecting infectious and endogenous danger signals [7,11-16]. In response, activated microglia and astrocytes produce inflammatory mediators like cytokines and chemokines, which critically modulate the course of CNS diseases. Therefore, understanding the mechanisms of glial activation is crucial to understanding the pathophysiology of the CNS.

Recent studies suggest that the chemokine receptor CXCR3 and the corresponding chemokine ligands CXCL9, CXCL10 and CXCL11 are expressed during CNS diseases and modulate glial functions like migration of microglia or cytokine synthesis [17-26].

CXCR3 ligands in CNS diseases are produced by infiltrating lympho- and monocytes but also by resident glial cells on stimuli such as TLR ligands or pro-inflammatory cytokines like IFN-gamma and TNF alpha [27-30]. The corresponding chemokine receptor CXCR3 can be differentially activated by the chemokine ligands CXCL9, CXCL10 and CXCL11 [31-35]. CXCR3 mediates distinct functions like integrin activation, cytoskeletal changes, suppression of angiogenesis or chemotactic migration [31,36-39]. Activated T cells and NK cells are well established to express CXCR3 at high levels [40]. Moreover, recent data argue for the functional existence of CXCR3 on microglia [23,41,42] and astrocytes [41,43]. CXCR3 signaling has been mostly investigated during infectious, bacterial and autoimmune diseases of the CNS [18,24,44-52] where immune cells infiltrate the CNS. However, the impact of CXCR3 on glial function without a significant influx of immune cells is not well characterized and only addressed by a few studies [23,53].

Cuprizone intoxication in C57BL/6 mice leads to primary oligodendrocyte apoptosis accompanied by an overwhelming activation of microglia and astrocytes in the absence of lymphocyte influx from the blood [54-56]. Using the cuprizone model we wanted to determine the impact of the CXCR3 chemokine system on endogenous glial activation.

Indeed, our data demonstrate that CXCR3 critically modulates the activation of glial cells by molecular and morphological criteria during cuprizone-induced toxic demyelination.

## Methods

### Animals and administration of cuprizone

Toxic demyelination was induced in 8-week-old wild type C57BL/6 (WT, Charles River) and CXCR3-deficient mice (CXCR3-/-; *B6.129P2-Cxcr3tm1Dgen/J*, Jackson Laboratory) fed with 0.2% (w/w) cuprizone (bis-cyclohexanone oxaldihydrazone, Sigma-Aldrich, München, Germany). Cuprizone was mixed into a powdered ground standard rodent chow. Before cuprizone administration, all mice of the study were adapted to powdered normal chow for one week. Cuprizone diet was then maintained for five weeks following four additional days on normal chow, representing the stage of CNS-remyelination. As a marker for the clinical outcome of cuprizone administration, the body weight of all mice was monitored twice a week. A total of 4 to 5 WT and CXCR3-/- mice were analyzed at each of the four time points of the experiment (0, 3, 5, and 5 weeks + 4 additional days normal chow/ 5.5 weeks). Animals were deeply anesthetized and transcardially perfused with ice cold phosphate buffered saline (PBS). Brains were removed immediately and cut along the sagittal midline. Brain hemispheres were fixed in 4% paraformaldehyde for 12 hours following paraffin-embedding. The remaining hemisphere was further processed for flow cytometry and quantitative PCR analysis.

### Routine histology and immunohistochemistry

A total of 8-μm thick paraffin-embedded sections were rehydrated in graded ethanol series after deparaffination in xylene and stained with hematoxylin and eosin (H&E) or Luxol fast blue (LFB) for routine histological analysis and myelin evaluation. For immunohistochemistry, sections were blocked with PBS containing 5% bovine serum albumin (BSA fraction V, PAA) for 30 min. For the detection of myelin proteins, antibodies specific for the proteolipid protein (PLP, Serotec; 1:200) and myelin basic protein (MBP, Dako; 1:200) were incubated for one hour at room temperature. Microglia cells were labeled using biotinylated tomato lectin (Sigma-Aldrich; 1:50). After washing in PBS, primary antibody treated sections were incubated with the appropriate biotinylated secondary antibody (Axxora; 1:200) before horseradish peroxidase-coupled streptavidin was applied (Axxora; 1:200). The signal was visualized by NovaRED color reagent (Vector Laboratories), according to the manufacturer’s instructions. To further analyze activated microglia, immunofluorescence stainings for ionized calcium binding adaptor molecule 1 (Iba1, Wako; 1:500) and macrosialin (CD68, Serotec; 1:500) combined with MBP antibody (Dako; 1:200) were applied. Sections were boiled with citric buffer (pH 6.0) for 10 min and washed with PBS containing 0.1% Triton-X 100 before standard blocking and antibody incubation procedures. After washing in PBS, Alexa-555 and Alexa-488 fluorescence-conjugated secondary antibodies (Invitrogen; 1:200) were used to visualize the primary antibody. Sections were counterstained with DAPI (Sigma-Aldrich) and mounted with fluorescent mounting medium (Dako). Conventional and immunofluorescence-stained sections were examined using the Eclipse E800 bright field and fluorescence microscope (Nikon). Bright field images and monochrome fluorescent images were acquired using a SPOT FLEX 64 Mp Shifting Pixel CCD-camera (model #15.2, Diagnostic Instruments Inc., Visitron Systems GmbH) and SPOT Basic Imaging software (Visitron Systems GmbH).

Quantification of lectin^+^ microglia and evaluation of the CD68^+^ and GFAP^+^ particle perimeter was performed using ImageJ threshold/analyze particle features. For high magnification images (CD68/Iba1 and GFAP/lectin immunofluorescence) sections were analyzed using a BX61 microscope equipped with a confocal disk scanning unit (Olympus).

### Flow cytometry analysis

The remaining half brains from WT and CXCR3-/- mice were placed into ice-cold Hank’s BSS buffer solution (PAA) and cut into small pieces. Samples were carefully disrupted and homogenized using a Potter-Elvehjem Tissue Grinder (10 ml; Wheaton), a needle (0.6 × 25) and a syringe (5 ml) before passing through a 70-μm cell strainer (BD Biosciences). After centrifugation, homogenates were dissolved in 75% Percoll (GE-healthcare). Subsequently, the homogenate was layered over with 25% Percoll and PBS. After centrifugation at 800xg for 25 minutes/4°C microglia were collected from the 25%/75% interface. For surface marker staining, the collected cells were washed in PBS, and blocked with CD16/CD32 (Fc block; eBioscience) antibody. Isolated cells were incubated with fluorochrome-conjugated antibodies (eBioscience) to detect CD3e (PerCP-Cy5.5), CD11b (APC), CD11c (PE-Cy7), CD45 (FITC), CD45 (eFluor 450), Ly6G (PerCP-Cy5.5), B220 (APC-eFluor 780) and NK1.1 (PE-Cy7). After washing, bound antibody was detected using a BD FACSCanto II (BD Biosciences) and data were analyzed using the flow cytometry software, FlowJo (TreeStar). Microglia were gated using forward and side scatter characteristics and identified by labelling with CD11b and CD45 according to their typical expression profile. CD11b^+^/CD45^dim^ microglia populations were further analyzed for the mean fluorescence intensity of CD11b and CD45. The percentage of CD11b^+^/CD45^high^ cells was expressed in comparison to CD11b^+^CD45^dim+high^ cells in the corresponding sample.

### Cytokine and chemokine mRNA determination by quantitative real-time polymerase chain reaction

Total RNA was isolated and purified from aliquots of homogenized brain samples using Trizol reagent (Sigma-Aldrich). Homogenates with Trizol were roughly shaken and disrupted using the Precellys homogenizer system (Bertin technologies). RNA quantity was determined using a NanoDrop 1000 (Peqlab). Up to 3 μg of total RNA was reverse-transcribed into cDNA by using SuperScript III Reverse Transcriptase (Invitrogen). Real-time quantitative PCR assays were performed on a StepOnePlus Real-Time PCR System (Applied Biosystems) using TaqMan™ Gene Expression Assays (Applied Biosystems) for *Gapdh, Plp1*, *Mbp*, *Cnp*, *Cxcl9*, *Cxcl10*, *Tnf*, *Il6*, *Ifng*, *Ccl2*, *Ccl3*, *Ccl5*. Samples were analyzed simultaneously for *Gapdh* mRNA as the internal control. Each sample was assayed in duplicate, normalized to the internal control and data was presented as copies of mRNA/*Gapdh* (means ± SEM).

### Statistical analysis

For all statistical analysis, differences between groups were tested using GraphPad Prism 5 (GraphPad Software Inc). One-way-ANOVA followed by Tukey’s post hoc test was applied to evaluate differences between studied groups. Statistical significance has been considered with a *P* value ≤0.05. All data are given as arithmetic means ± SEM.

## Results

### CXCR3-deficiency reduces clinical symptoms and weight loss during cuprizone feeding

As previously shown, cuprizone feeding triggers growth retardation and a decline of body weight in mice [57,58]. We documented the body weight development and phenotypic signs for sickness in CXCR3-/- and WT mice during the course of cuprizone feeding.

We found a scruffy coat combined with a hunched posture and ocular/nasal discharge as clinical symptoms for cuprizone intoxication during the first three weeks of the cuprizone-diet in most of the WT mice. These findings were less overt in CXCR3-/- mice. We also observed an overall higher locomotor activity of CXCR3-/- mice compared to WT controls.

**Figure 1:**
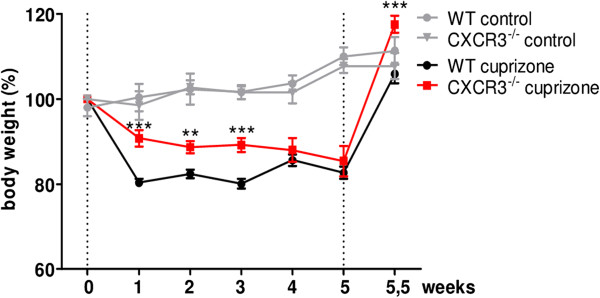
**Cuprizone administration induces a less overt body weight decline in CXCR3-/- compared with wild type (WT) mice.** From 8 weeks of age, WT and CXCR3-/- mice were fed with 0.2% cuprizone for five weeks (dotted grid lines) with a subsequent four days of recovery to normal chow (5.5 weeks). The body weights were monitored twice a week and plotted in comparison with values of control animals fed with powdered normal chow (gray symbols). During the initial three weeks of cuprizone feeding, CXCR3-/- animals showed a significantly less pronounced body weight decline than WT mice (n = 20 per group; **P <0.01, ***P <0.001). Moreover, body weight recovery after offset of cuprizone feeding was found to be significantly improved in CXCR3-deficient mice after 5.5 weeks (n = 7 per group; ***P <0.001). Data represent mean ± SEM.

A rapid drop of body weight was detectable after the first week of cuprizone feeding, when the body mass of WT mice was reduced to 80.4 ± 0.9%, whereas in CXCR3-/- mice dropped to 90.8 ± 1.9% (Figure 1; ***P <0.001) of the initial weight (initial weight: WT 0 weeks: 19.4 ± 0.6 g, n = 20; CXCR3-/- 0 weeks: 18.8 ± 0.5 g, n = 20). During the subsequent two weeks of cuprizone feeding, the body weight remained at a significantly lower level in WT compared to CXCR3-/- mice (Figure 1; WT, 2 weeks: 82.4 ± 1.0% versus CXCR3-/-, 2 weeks: 88.7 ± 1.4%, **P <0.01, n = 20; WT, 3 weeks: 80.1 ± 1.1% versus CXCR3-/-, 3 weeks: 89.2 ± 1.7%, ***P <0.001, n = 20). Between week three and four, WT mice increased their weight by +5.5% to 85.6 ± 1.3% (n = 14), whereas CXCR3-/- mice remained at 88.0 ± 2.8% (n = 14) of their initial weight. From week four to five of cuprizone administration no significant differences in the mean body masses were detectable between both genotypes. Withdrawal of cuprizone after five weeks resulted in rapid body weight recovery in WT and CXCR3-/- mice. The gain of body weight during withdrawal of cuprizone was significantly higher in mice deficient for CXCR3 compared to WT animals (Figure 1; WT 5.5 weeks: 105.9 ± 2.2%; CXCR3-/- 5.5 weeks: 117.7 ± 1.9%; *P <0.05).

### CXCR3 is critical for the spatial distribution and activation of microglia

As previously indicated, CXCR3 potentially influences the functional properties of microglia during pathological states of the CNS [23,53]. Here, we characterized the impact of CXCR3 deficiency on microglia activation and accumulation during cuprizone treatment. First, we detected activated microglia in different brain regions: corpus callosum (Cc), cerebellum (Cb) and thalamus (Th) using tomato lectin staining, which was quantified as a degree for microglial accumulation and activation.

**Figure 2:**
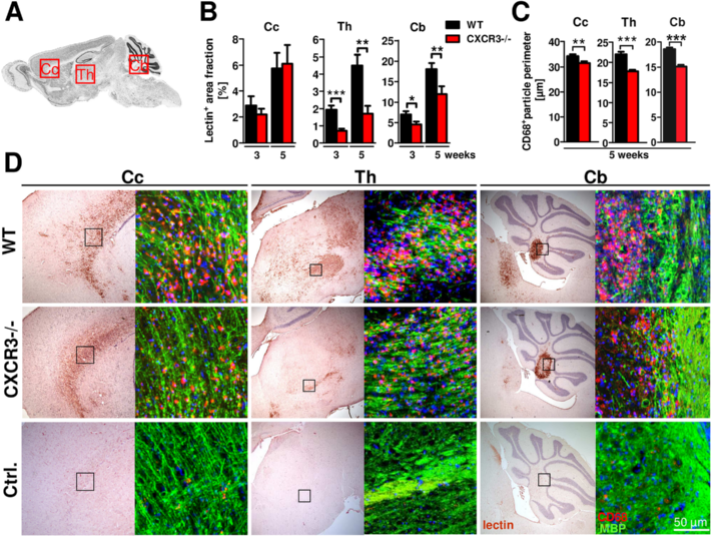
**Diminished microglia cell numbers and extent of CD68**^**+ **^**content in CXCR3-/- cuprizone brains. A**. Sagittal brain sections of wild type (WT) and CXCR3-/- were analyzed for the localization of activated microglia at regions of the corpus callosum (Cc), thalamus (Th) and cerebellum (Cb). **B**. Analysis of serial sagittal brain sections stained for tomato lectin documents a significant reduction of activated microglia in cuprizone-fed CXCR3-/- mice compared to WT animals (n = 10 to 14 at equal sections, time point and genotype, *P <0.05, **P <0.005, ***P <0.001). **D**. These observations were made within regions of the thalamus (Th) and cerebellum (Cb), but were not found in the white matter of the frontal corpus callosum (Cc) of CXCR3-/- brains. Besides the reduced lectin^+^ area fraction in the cerebellum of CXCR3-/- mice, we also found a differential spatial distribution of microglia surrounding the cerebellar nuclei together with a consistent cluster at the vestibular nuclei of the medulla after 5 weeks (D, WT Cb versus CXCR3-/- Cb). {AU Query: Please define ‘D’ from the previous sentence so readers know what this abbreviation represents.} Because most overt differences of microglia accumulation between CXCR3-/- and WT were visible after 5 weeks of cuprizone diet, we further investigated microglia using double fluorescence immunolabeling for CD68 (red) and MBP (green) (Myelin basic protein). **C**. At the designated insets (D) we analyzed the CD68^+^ content in both genotypes and found significantly reduced particle perimeters in all studied central nervous system (CNS) regions in CXCR3-/- compared to WT brains (n ≥1,900 CD68^+^ particles per genotype, **P <0.01, ***P <0.001). Data represent mean ± SEM.

Compared to WT, an attenuated amount of lectin^+^ signal was detected in the cerebellum of CXCR3-/- animals (Figure 2B, Cb; WT, 3 weeks: 7.0 ± 0.8% versus CXCR3-/-, 3 weeks: 4.6 ± 0.7%, *P <0.05, lectin^+^ area fraction; WT, 5 weeks: 18.1 ± 1.4% versus CXCR3-/-, 5 weeks: 12.0 ± 1.9%). Moreover, strikingly diminished lectin staining was found in the thalamus (Th), the midbrain and the hypothalamus of CXCR3-/- mice (Figure 2B, Th; WT, 3 weeks: 1.9 ± 0.2% versus CXCR3-/- 3 weeks: 0.7 ± 0.1%, ***P <0.001; WT, 5 weeks: 4.5 ± 0.6% versus CXCR3-/- 5 weeks: 1.7 ± 0.5%, **P <0.01, lectin^+^ area fraction).

Together with attenuated microglia accumulation, microglia showed a differential spatial distribution in the cerebellum of CXCR3-/- animals. Consistently, we found a rim-like formation of microglia in the deep cerebellar white matter (Figure 2D, Cb; 5 weeks CXCR3-/-) in CXCR3 deficient brains. In contrast, WT mice displayed a dense and even distribution of microglia in this area. In difference to CXCR3-/- animals, we also found widespread lectin^+^ cells in the medulla in WT mice (Figure 2D, Cb; WT 5 weeks).

However, at the stage of acute demyelination after three and five weeks, we observed comparable numbers of lectin^+^ microglia in the corpus callosum of WT and CXCR3-/- mice (Figure 2B, D; Cc).

**Figure 3:**
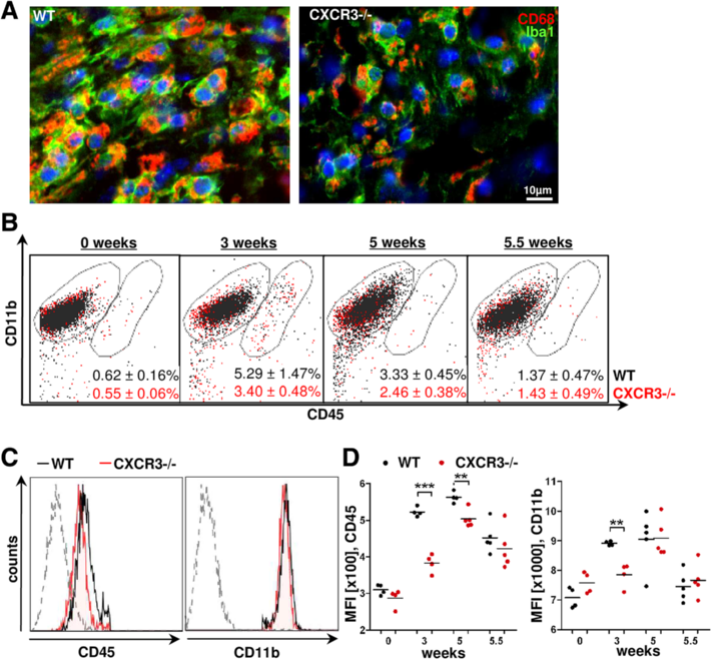
**CXCR3-deficient microglia display diminished levels for CD45 and CD11b during cuprizone diet. A**. E*x vivo-*prepared microglia of both genotypes were phenotyped using flow cytometry. **B**. Brain homogenates from wild type (WT) and CXCR3-/- mice after 3, 5, and 5.5 weeks of the cuprizone diet (*n* = 4 to 5 animals per group) were prepared and microglia subsequently analyzed for CD45 and CD11b expression. Using CD11b/CD45 gating, the majority of microglia were found in a distinct isolated CD11b^+^/CD45^dim^ population in WT and CXCR3-/- animals (left encircled population). A small population of CD11b^+^/CD45^high^ cells was slightly increased in WT compared to CXCR3-/- mice after 3 weeks (right encircled population, percentage of CD11b^+^/CD45^high^ cells is given below, mean ± SEM). **C**. Flow cytometry histogram overlays show representative CD45/CD11b expression from freshly isolated microglia (CD11b^+^/CD45^dim+high^ in WT (black line) and CXCR3-/- (red line) cuprizone-fed mice after 3 weeks. The dashed histogram represents the isotype control. Histograms were gated on microglial population according to forward/side scatter profile. CXCR3-/- animals displayed reduced surface expression levels for CD45 and CD11b compared with WT levels. **D**. Statistical analysis of the CD45 and CD11b level within the CD11b^+^/CD45^dim^ microglia population of WT (black dots) and CXCR3-/- (red dots) mice over the course of the cuprizone experiment (mean fluorescence intensity, MFI). Fundamentally reduced MFI of CD45 (3 and 5 weeks) and CD11b (3 weeks) were documented in CXCR3-/- compared to WT microglia (**P <0.005, ***P <0.001).

Interestingly, microglia, localized in the white matter components exhibit larger and round-shaped CD68^+^ compartment in WT than in CXCR3-/- animals (Figure 2C, D; Cc). Quantification of CD68-positive particles, as a marker for microglial lysosomes, outlines a larger mean perimeter in WT compared to CXCR3-/- mice in the corpus callosum (Figure 2C, Cc; WT 5 weeks: 34.2 ± 0.7 μm (n = 1890) versus CXCR3-/- 5 weeks 31.58 ± 0.67 μm, n = 2286, **P <0.01), thalamus (Figure 2C, Th; WT 5 weeks: 22.0 ± 0.7 μm, n = 1442 versus CXCR3-/- 5 weeks: 18.5 ± 0.4 μm, n = 2200, ***P <0.001) and cerebellum (Figure 2C, Cb; WT 5 weeks: 18.6 ± 0.3 μm, n = 4265 versus CXCR3-/- 5 weeks: 15.2 ± 0.3 μm, n = 2444, ***P <0.001) of CXCR3-/- mice. To further define the alleviated microglia activation in CXCR3-/- mice we co-localized Iba1 and CD68 to differentiate activation by morphology and expression of a lysosomal antigen. In cuprizone-treated WT animals, tight clusters of activated Iba1+ microglia cells were visible. Microglia in these clusters displayed large CD68^+^ lysosomes, while in CXCR3-/- mice microglia distribution was loose and intracellular CD68^+^ lysosomes were smaller in size and less prominent (Figure 3A).

Taken together, during cuprizone treatment, diminished numbers and reduced activation of microglia were observed in CXCR3deficient mice compared to WT.

### Flow cytometry further confirms reduced microglial activation in cuprizone-treated CXCR3-deficient mice compared to wild type controls

The histological characterization of microglia in cuprizone-treated mice outlined a differential spatial distribution, accumulation and attenuated activation in CXCR3-/- mice compared to control animals (Figure 2B-D and Figure 3A). To further detect and substantiate differences in the microglial phenotype during cuprizone administration, we applied flow cytometry of brain homogenates and determined the CD11b and CD45 levels of microglia populations.

**Figure 4:**
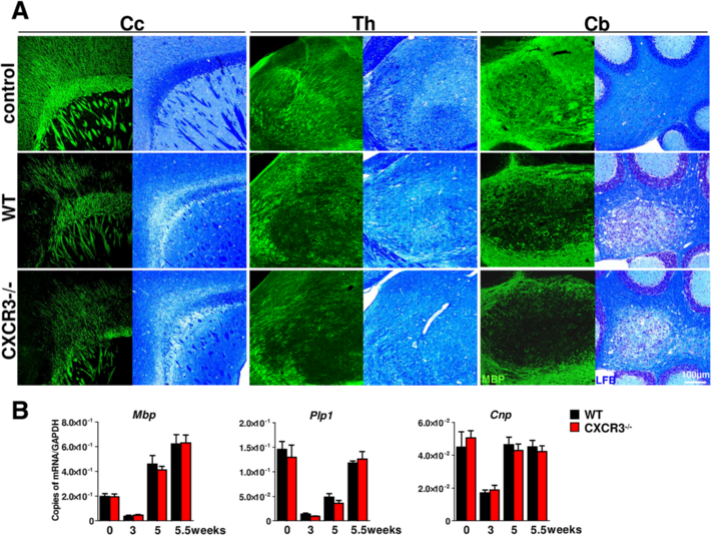
**Cuprizone intoxication induces a robust demyelination of different brain regions in wild type (WT) and CXCR3-/-mice. A**. Sagittal brain sections of WT and CXCR3-/- were analyzed for the degree of (de-)myelination at regions of the corpus callosum (Cc), thalamus (Th) and cerebellum (Cb) using immunofluorescence detection for myelin basic protein (MBP) and Luxol fast blue staining (LFB, bright field). Cuprizone-fed mice showed decreased MBP^+^ fluorescence signal in the frontal corpus callosum (Cc), thalamus (Th) and the nucleus of the cerebellum (Cb) in WT and CXCR3-/- mice after 5 weeks. No significant differences in the degree of demyelination were visible between WT and CXCR3-/- mice after LFB stainings. Pictures are representative of 4 to 5 mice per group at each location and condition. **B**. Quantification for *Mbp, Plp1* and *Cnp* transcripts using TaqMan assays document equal level in WT and CXCR3-/- mice at all dissected time points of the experiment. The most rapid drop of myelinogenic gene expression has been documented at the acute stage of demyelination after 3 weeks of cuprizone diet. Upregulation of the level of myelinogenic transcripts were found after withdrawal of cuprizone for 4 days (5.5 weeks). Data represent mean ± SEM, n = 4 to 5 for each genotype and time point.

As previously shown, CD45 levels can be helpful to discriminate infiltrating macrophages/ microglia (CD11b^+^/CD45^high^) or parenchymal microglia (CD11b^+^/CD45^dim^) [56,59]. We assessed the percentage of CD11b^+^ cells expressing diminished (dim) or high levels of CD45 (high). Initially, CD11b^+^/CD45^dim^ microglia comprised 99.4% of the CD11b^+^ cells in WT and CXCR3-/- animals (0 weeks, Figure 3). Throughout the experiment, these microglia were visible as a distinct cell population (Figure 4B, left encircled population in both genotypes). An overall low, but significantly higher percentage of CD11b^+^/CD45^high^ cells were found in both WT and CXCR3-/- animals upon cuprizone intoxication, peaking after 3 weeks of the study (Figure 4 B; WT 0 weeks: 0.62 ± 0.16% versus WT 3 weeks: 5.29 ± 1.47%, *P <0.05; CXCR3-/- 0 weeks: 0.55 ± 0.06% versus CXCR3-/- 3 weeks: 3.40 ± 0.48%, **P <0.01). Although, the populations of CD11b^+^/CD45^high^ microglia/ macrophages were slightly increased after 3 and 5 weeks in WT animals, this difference did not reach a significant t level when compared to CXCR3-/- mice.

We further analyzed the CD11b^+^/CD45^dim^ microglia population and found strongly reduced surface expression of CD45 on CXCR3-/- compared to WT microglia after three and five weeks of cuprizone treatment (Figure 3B-D). After three weeks of cuprizone ingestion, the reduction of CD45 and CD11b molecules on CXCR3-/- microglia was most intense compared to WT mice (Figure 3C; CD45, WT: 522.8 ± 6.5 versus CXCR3-/-: 382.3 ± 12.7, ***P <0.001; CD11b WT: 8912 ± 35.9 versus CXCR3-/-: 7845 ± 202.0, MFI). After five weeks, the expression of microglial CD45 in CXCR3-/- mice was still significantly lower than in WT mice (Figure 3C; CD45, WT 3 weeks: 562.8 ± 7.4 versus CXCR3-/- 3 weeks: 503.6 ± 10.5, **P <0.005, MFI) whereas the CD11b expression reached the corresponding WT level.

The detection of other CD45^+^ leukocytes like neutrophils (Ly6-G), T cells (CD3e) and Nk cells (Nk 1.1) did not lead to evaluable data. We found very low quantities of other CD45-positive cells than the CD11b^+^/ CD45^+^ population. The percentage of CD11b-negative CD45^+^ events continuously lay below 1.3% in WT and CXCR3-/- animals (data not shown).

In summary, CD11b^+^/CD45^dim^ CXCR3-/- microglia displayed strongly diminished total level of the activation markers CD45 and CD11b during cuprizone administration when compared to WT controls. However, we could not detect significant differences in the composition of CD11b^+^/CD45^dim^ and CD11b^+^/CD45^high^ microglia/ macrophages between WT and CXCR3-deficient mice throughout the cuprizone study.

### Astrocyte activation by morphological criteria is reduced in cuprizone-treated CXCR3-/- mice compared to wild type controls

**Figure 5:**
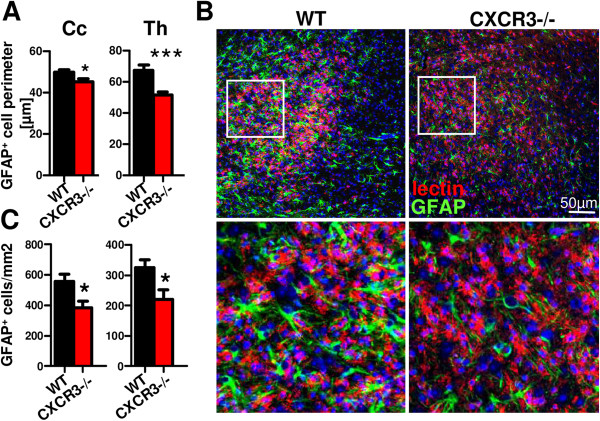
**Astrocytes exhibit diminished morphological activation in CXCR3-/- animals. A**. By using morphometric analysis of equal sections in wild type (WT) and CXCR3-/- mice, we noticed a significant increase of the mean astrocyte perimeter in corpus callosum (Cc) and thalamus (Th) regions after five weeks of cuprizone diet (n ≥ 700 GFAP^+^ cells per genotype and central nervous system (CNS) region, *P <0.05, ***P <0.001). **B**. Combined immunofluorescence staining for astrocytes-specific GFAP and microglia using tomato lectin demonstrates reduced cellular extensions and less pronounced hypertrophic GFAP^+^ astrocytes in CXCR3-deficient brains. These findings were detectable close to zones of microglia activation in the Cc (not shown) and Th (overview together with enlarged insets below). **C**. Quantification of GFAP^+^ cells in both regions of interest indicates increased numbers in WT compared to CXCR3-deficient mice (*P <0.05). Data represent mean ± SEM.

Subsequently, we prepared immunofluorescence stainings for glial fibrillary acidic protein (GFAP) specific antibody combined with lectin to localize reactive astrogliosis within regions of microglia activation. Hypertrophic astrocytic cell bodies were found in close proximity to the corpus callosum (Cc) and within the thalamus (Th) of WT, but were less pronounced in CXCR3-/- mice. We quantified the extent of astrocytic activation in terms of GFAP-positive cell body perimeters and found reduced values in CXCR3-/- animals within the Cc and Th regions investigated (Figure 5A; WT Cc: 49.9 ± 1.3 μm, n = 1672 versus CXCR3-/-Cc: 45.4 ± 1.3 μm, n = 1303; *P <0.05). This effect was most prominent in the thalamic region and in brain areas with high microglia accumulation in WT and CXCR3-/- mice (Figure 5A; WT Th: 67.5 ± 3.2 μm, n = 1220 versus CXCR3-/- Th: 51.4 ± 2.3 μm, n = 930; ***P <0.001). Substantial overlapping of neighboring astrocyte processes with confluent cellular signals were detected in WT but not in CXCR3-/- animals (Figure 5B, Th; insets of original magnification). In all three regions of interest, GFAP + cells were quantified using sections of both genotypes from corresponding brain regions. In thalamic regions and the corpus callosum, we could find significantly reduced numbers of GFAP^+^ cells in CXCR3-/- compared to WT animals (Figure 5C; WT Th: 325 ± 25 versus CXCR3-/- Th: 219 ± 33 and WT Cc: 556 ± 48 versus CXCR3-/- Cc: 384 ± 43 *P <0.05, cells/mm2).

### Attenuated levels of proinflammatory transcripts in CXCR3-deficient mice correlate with diminished accumulation and activation of microglia and astrocytes

To correlate the observation of diminished glial activation with changes in the local cytokine milieu, we compared the brain levels of key inflammatory cytokines during cuprizone treatment of CXCR3-deficient and WT mice.

**Figure 6:**
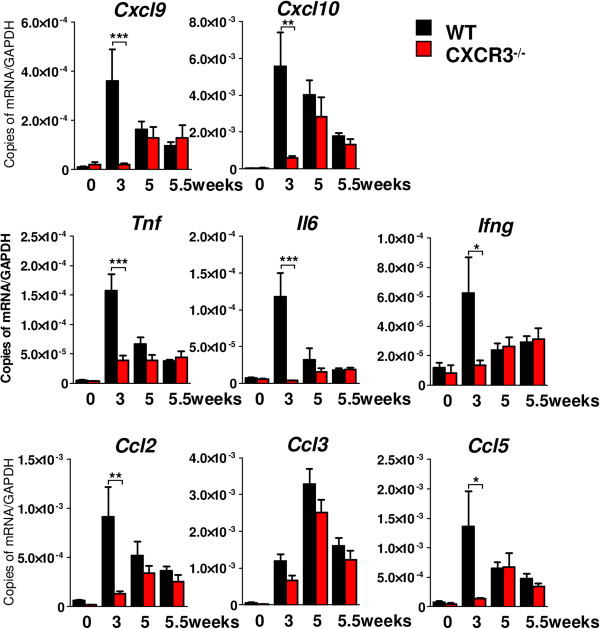
**Substantially reduced transcripts for proinflammatory cytokines and chemokines in CXCR3-/- brains.** Total RNA was isolated from freshly prepared brain homogenates using Trizol before cDNA was synthesized using Superscript III enzyme. TaqMan gene expression assays revealed a strongly reduction of various inflammatory transcripts after three weeks of cuprizone diet in CXCR3-deficient brain. Note the early induction of *Cxcl9, Cxcl10, Ccl5* and *Ccl2* mRNA expression levels after three weeks of cuprizone diet, correlating with the high levels of *Tnf, Il6* and *Ifng* in WT brains. In contrast, in CXCR3-/- brains, transcript levels only slightly increased compared to control levels (0 weeks). Values were normalized against a housekeeping gene *Gapdh* (*P <0.05, **P <0.005, ***P <0.001, mean ± SEM, n = 4 to 5 for each genotype and time point).

Consistent with the histopathologic features, the induction of various tested cytokines and chemokines was most striking after three weeks in WT animals. At that time point we could detect no (*IL6, Ifng*, *Cxcl9* and *Ccl5*) or only minimal (*Tnf, Ccl2, Ccl3* and *Cxcl10*) upregulated transcripts in CXCR3-/- mice compared to untreated controls. After 3 weeks, the CXCR3 ligands *Cxcl9* and *Cxcl10* were found highly upregulated in WT (Figure 6, *Cxcl9*: WT ctrl. 1.1 ± 0.2 × 10^-5^ versus WT 3 weeks 3.6 ± 1.3 × 10^-4^, *Cxcl10*: WT ctrl. 4.2 ± 0.3 × 10^-4^ versus WT 3 weeks 5.6 ± 1.8 × 10^-3^, copies of mRNA/*Gapdh*). Simultaneously, transcripts for the pro-inflammatory cytokines *Tnf* (3 weeks, WT: 1.6 ± 0.3 × 10^-4^ versus CXCR3-/-: 3.9 ± 0.9 × 10^-5^, copies of mRNA/*Gapdh*), *Il6* (3 weeks, WT: 1.2 ± 0.3 × 10^-4^ versus CXCR3-/-: 3.8 ± 0.4 × 10^-6^, copies of mRNA/*Gapdh*) and *Ifng* (3 weeks, WT: 6.2 ± 2.5 × 10^-5^ versus CXCR3-/-: 1.4 ± 3.6 × 10^-6^, copies of mRNA/*Gapdh*) peaked in WT but were strongly reduced in CXCR3-/- animals.

Decreased quantities for *Tnf, IL6, Ifng*, *Cxcl9*, *Cxcl10, Ccl2, and Ccl5* transcripts were detectable throughout all later time points in WT brains (Figure 6, 5 and 5.5 weeks), ultimately reaching the levels in CXCR3-/- animals. In contrast to all other targets, we only found *Ccl3* with its highest level after 5 weeks in WT and CXCR3-/- animals.

The findings of the qRT-PCR analysis outline a striking attenuation of proinflammatory cytokines in CXCR3-deficient mice compared to WT animals during cuprizone feeding.

### Cuprizone-mediated demyelination is not significantly altered in CXCR3-deficient mice

Cuprizone ingestion induces a highly reproducible and robust demyelination of multiple brain regions in WT mice. We applied Luxol fast blue (Figure 4 A, LFB) routine stainings and immunohistochemistry (Figure 4A, myelin basic protein, MBP) to detect demyelination in WT and CXCR3-/- brains at all time points examined. By using microscopy and image analysis of serial sections at various CNS regions, no differences in the degree of demyelination were documented between WT and CXCR3-/- after five weeks of cuprizone administration (Figure 4A). At this stage of chronic demyelination in WT and CXCR3-/- mice, robust demyelination was monitored in the corpus callosum, thalamus/hypothalamus and the cerebellum.

Furthermore, we performed qPCR of myelin basic protein (*Mbp*), proteolipid protein (myelin) 1 (*Plp1*) and 2',3'-cyclic nucleotide 3' phosphodiesterase (*Cnp*), which indicates oligodendrocytic gene transcription of myelin protein components (Figure 4B). Downregulation of these mRNA species clearly precedes the onset of significant demyelination after three weeks of the cuprizone study. These changes were indistinguishable between WT and CXCR3-/- mice (Figure 4B; *Mbp*, *Plp1* and *Cnp* level in WT 3 weeks versus CXCR3-/- 3 weeks). After five weeks expression of *Cnp* returned to normal control values (Figure 4B, WT 5 weeks: 4.6 ± 0.5 × 10^-2^ versus WT 0 weeks: 4.5 ± 0.1 × 10^-2^ and CXCR3-/- 5 weeks: 4.3 ± 0.4 × 10^-2^ versus CXCR3-/- 0 weeks: 4.5 ± 0.1 × 10^-2^; copies of mRNA/*Gapdh*) or were found higher in case of *Mbp* transcripts (Figure 4B, WT 5 weeks: 4.6 ± 0.7 × 10^-2^ versus WT 0 weeks: 20.0 ± 0.2 × 10^-2^; copies of mRNA/*Gapdh*). Again, no significant differences were detectable between WT and CXCR3-/- animals. We could also demonstrate the reappearance of *Plp1* expression during late stages of acute demyelination at five weeks, whereas levels did not reach the corresponding control level (Figure 4B, WT 5 weeks: 14.6 ± 0.2 × 10^-2^ versus WT 0 weeks: 4.8 ± 0.7 × 10^-2^ and CXCR3-/- 5 weeks: 4.0 ± 0.6 × 10^-2^ versus CXCR3-/- 0 weeks: 12.9 ± 0.3 × 10^-2^; copies of mRNA/*Gapdh*, mean ± SEM). Withdrawal of cuprizone diet did not alter the levels for myelin specific transcripts detectable between WT and CXCR3-/- animals.

## Discussion

Glial activation is a key feature of neuroinflammatory and neurodegenerative diseases. Depending on their functional state, glial cells can have both a beneficial and a harmful impact on the course of neurological diseases. Understanding which factors control and modulate glial activation is critical to understand these diseases and to develop novel therapeutic approaches. Here, we examined the impact of the chemokine receptor CXCR3 on glial activation in cuprizone-induced demyelination as a prototypical model for an endogenous activation of both microglia and astrocytes without a major influx of hematogenous immune cells like lymphocytes or monocytes [56,59]. This is in contrast to most of the previous studies examining CXCR3 in the context of neuroinflammatory models with a high influx of hematogenous leukocytes into the brain tissue.

First, we observed that the clinical phenotype and the weight loss accompanied with cuprizone intoxication were attenuated in CXCR3-deficient mice when compared to wild type animals. Although the clinical phenotype during cuprizone feeding is well described [57,58], the pathophysiological mechanisms are not yet defined. Therefore, we can only speculate how CXCR3 deficiency leads to the attenuated phenotypical changes. The observed changes in glial activation and cytokine profile as discussed later might be an indicator for similar changes in peripheral organs like the liver, which in turn become reflected by clinical symptoms and weight loss.

When examining the glial phenotype, we examined whole sagittal brain sections because the dynamics of cuprizone-induced demyelination and glial activation differ widely between the brain areas [60-62]. We characterized microglial activation by lectin histochemistry and found differences in microglial activation for all examined brain regions. The differences in areas like the cerebellum or the thalamus were striking and highly significant. Notably, the effect of CXCR3 on microglial accumulation and glial activation was less prominent in the corpus callosum, although cuprizone-induced myelination is most distinct in this area. These regional differences of the effect of CXCR3-deficiency might be explained with the different state of demyelination in these areas. Very extensive demyelination as observed in the corpus callosum could lead to a maximal microglial activation, which cannot be attenuated by the loss of one modulating factor like CXCR3. Still, these findings demonstrate the potent effect of CXCR3 on glial activation in the examined model. The differences in microglial activation were further substantiated by co-localization of the microglial activation marker CD68, which was much lower expressed in microglia from CXCR3-deficient mice, and by flow cytometry. Here, we found most prominent differences in CD45 and CD11b after three weeks of cuprizone feeding.

The modulating effects of CXCR3 were not restricted to microglial cells. GFAP staining and analysis of GFAP^+^ cells revealed that GFAP^+^ astrocytes were less frequent and less activated by morphological criteria in CXCR3-deficient mice. However, we cannot discriminate if this observation is directly induced by CXCR3 deficiency or the consequence of reduced microglial activation. Skripuletz *et al*. demonstrated in a recent study that a lack of astrocytes during cuprizone feeding results in less demyelination and less microglial activation and found evidence that this effect is mediated by a lack of astrocytic CXCL10 [63]. In regard to our study, we cannot exclude that the changes in microglial activation we observe are at least partly caused by a reduced synthesis of CXCL10 by CXCR3-deficient astrocytes. However, a study examining the effect of chronic expression of cerebral CXCL10 could not detect an impact of CXCL10 on microglial activation [64]. As astrocytes and microglia intimately interact and both carry the CXCR3 receptor, it is likely that the lack of CXCR3 on microglia and astrocytes has a synergistic effect on the activation of the cell types [65].

To evaluate the impact of the reduced glial activation on the inflammatory milieu during cuprizone treatment, we determined the RNA levels of key inflammatory cytokines and chemokines. Corresponding to the activation time-course revealed by flow cytometry, we found a striking attenuation of all inflammatory molecules examined. This was most prominent for prototypical proinflammatory cytokines like TNFα or the chemokine CCL2. Interestingly, the RNA levels of the CXCR3 ligands CXCL9 and CXCL10 were drastically reduced and comparable to untreated controls in CXCR3-deficient mice. This might argue for an auto- or paracrine loop in which CXCR3-activation increases the production of CXCR3 ligands in glial cells.

Lastly, we characterized the impact of CXCR3 on the course of de- and remyelination in different areas of the brain. Despite the striking changes in the glial activation and cytokine expression, we could not detect a significant change in the course of de- and remyelination. Neither histological evaluation nor the course of the molecular markers Mbp, Plp1 or Cnp revealed differences of de- and remyelination between CXCR3-competent and CXCR3-deficient mice. Although there is some evidence that oligodendrocytes express a functional CXCR3 receptor [66,67], the lack of differences in demyelination argues strongly against a direct modulatory effect of CXCR3 on the extent of oligodendrocyte damage in this model. During remyelination after cuprizone-induced demyelination, oligodendrocyte precursor cells become recruited, proliferate and have to undergo maturation [68,69]. Although we did not analyze the oligodendrocyte population in extent, we could not observe differences in the increase of myelin-related RNA levels after cessation of cuprizone feeding, nor in the amount of remyelination. This argues, like discussed for demyelination, against a function of CXCR3 in remyelination after cuprizone demyelination.

## Conclusions

In conclusion, our findings argue for a major role of CXCR3 in the endogenous activation of glial cells induced by cuprizone-mediated demyelination. This provides further evidence for an important role of this chemokine receptor in modulating the function of microglia and astrocytes.

## Abbreviations

Cb: cerebellum; Cc: corpus callosum; GFAP: glial fibrillary acidic protein; H&E: hematoxylin and eosin stain; LFB: Luxol fast blue staining; MBP: myelin basic protein; MFI: mean fluorescent intensity; qRT-PCR: quantitative real-time polymerase chain reaction; Th: thalamus; WT: wild type.

## Competing interests

The authors declare that they have no competing interests.

## Authors’ contributions

MK performed histology/histomorphometry, analyzed the data and wrote the manuscript. SS carried out the experiment, acquired clinical data, processed tissue (for histology) and managed real-time PCR analysis. JZ performed flow cytometric analysis and participated with MTH and MM in the experimental design. ME was involved in histological staining procedures like LFB and the analysis of these procedures. MM wrote parts of the manuscript, was responsible for conception and coordination and discussed and interpreted the acquired data. All authors read and approved the final version of this manuscript.
